
JOURNAL INFORMATION
==============================
NLM Title Abbreviation: Buffalo Med Surg J
NLM Journal ID: 0004275

Buffalo Medical and Surgical Journal

ISSN: 1040-3825
Publisher: Biomedical Journal Digitization Project

ARTICLE INFORMATION
==============================
PMCID: PMC9488413
Article version: 1
Subjects: Book Reviews

Philosophy in Homeopathy

Print publication date: 1890 Oct
Volume: 30
Volume Series: New Ser
Issue: 3
First page: 192
Last page: 192
License: This work is free of known copyright restrictions. For more information, please see PMC Back Issue Digitization.
License URL: https://creativecommons.org/publicdomain/mark/1.0/

==============================

F. H. P.

---

PHILOSOPHY IN HOMEOPATHY, addressed to the Medical Profession and to the General Reader. By CHARLES S. MACK, M. D., Professor of Materia Medica and Therapeutics in the Homeopathic Medical College of the University of Michigan at Ann Arbor. Chicago: Gross & Delbridge. 1890. Pp. 174; cloth, 8vo.

The author of this book looks on one side of the shield and tells us just what he sees from his standpoint. There is an earnestness in his style which we wish was devoted to matter which carried conviction.

But he is too firm a believer to convince. He is one of the few men who elevates a human system to a Divine law. He says: "I believe that homeopathy is without flaw." Believing this, we do not see how he could very well be anything but a homeopathist.

The publishers' work is well done. J. W. P.

FOR SALE.—"Medical and Surgical History of the Rebellion." Six volumes. In complete order. Price on application to

THEO. G. LEWIS,

15 Court street, Buffalo, N. Y.

### NOTICE TO CONTRIBUTORS.

We are glad to receive contributions from every one who knows anything of interest to the profession. Articles designed for publication in the JOURNAL should be handed in before the first day of the month.

The Editors are not responsible for the views or opinions of contributors.

All communications should be addressed to

284 FRANKLIN STREET,

BUFFALO, N. Y.
